# Advancing Brain Health Naturally: β-Caryophyllene and Xanthohumol as Neuroprotective Agents

**DOI:** 10.3390/molecules30183702

**Published:** 2025-09-11

**Authors:** Stanislava Ivanova, Zoya Dzhakova, Velislava Todorova, Radka Boyuklieva, Plamen Simeonov, Plamen Katsarov

**Affiliations:** 1 Department of Pharmacognosy and Pharmaceutical Chemistry, Faculty of Pharmacy, Medical University of Plovdiv, 4002 Plovdiv, Bulgaria; 2 Research Institute, Medical University of Plovdiv, 4002 Plovdiv, Bulgaria; 3 Department of Pharmaceutical Technology and Biopharmacy, Faculty of Pharmacy, Medical University of Plovdiv, 4002 Plovdiv, Bulgaria

**Keywords:** neuroprotection, β-caryophyllene, natural medicine, xanthohumol, neurodegenerative diseases, nanotechnology

## Abstract

Neurodegenerative diseases (NDDs) represent a class of incurable and progressive disorders characterized by the gradual degeneration of the structure and function of the nervous system, particularly the brain and spinal cord. A range of innovative therapeutic approaches is currently under investigation, such as stem cell-based therapies, gene-editing platforms such as CRISPR, and immunotherapies directed at pathogenic proteins. Moreover, phytochemicals such as β-caryophyllene and xanthohumol have demonstrated significant neuroprotective potential in preclinical models. These natural agents exert multifaceted effects by modulating neuroinflammatory pathways, oxidative stress responses, and aberrant protein aggregation—pathological mechanisms that are central to the development and progression of neurodegenerative disorders. Recent investigations have increasingly emphasized the optimization of the pharmacokinetic properties of β-caryophyllene and xanthohumol through the development of advanced drug-delivery systems, including polymer- and lipid-based nano- and microscale carriers. Such advancements not only enhance the bioavailability and therapeutic potential of these phytochemicals but also underscore their growing relevance as natural candidates in the development of future interventions for neurodegenerative disorders.

## 1. Introduction

Neurodegenerative diseases (NDDs) represent a class of incurable and progressive disorders characterized by the gradual degeneration of the structure and function of the nervous system, particularly the brain and spinal cord [1]. These conditions often lead to debilitating cognitive, behavioral, and motor impairments, significantly affecting the quality of life and placing a considerable burden on caregivers and healthcare systems. As life expectancy increases globally, the prevalence of neurodegenerative disorders is rising, prompting urgent research into their causes, classification, and treatment. Neurodegenerative disorders affect a substantial portion of the global population. Although advancing age is one of the most significant risk factor associated with the onset of NDDs, accumulating evidence suggests that the interplay between genetic susceptibility and environmental exposures also substantially contributes to disease pathogenesis and progression [1].

Several classifications exist regarding neurodegenerative diseases [2,3]. According to the affected protein pathology Alzheimer’s disease (AD) is associated with accumulation of amyloid-β and hyperphosphorylated tau, the Parkinson’s disease (PD) is associated with α-synuclein aggregates in Lewy bodies, the amyotrophic lateral sclerosis (ALS) is associated with TDP-43 or SOD1 protein dysfunction and Huntington’s disease (HD) is associated with mutant huntingtin protein due to CAG triplet repeat expansion [4].

Regarding Clinical Manifestation the neurodegenerative disorders could be classified as cognitive disorders (AD, frontotemporal dementia) [5], motor disorders (PD, ALS, HD) [4,6], and mixed disorders (dementia with Lewy bodies, corticobasal degeneration) [7,8]. Parkinson’s disease is considered the second most prevalent progressive neurodegenerative disorder (ND) [9]. As mentioned above, this ND could be regarded as motor disorder. Its motor symptoms—include tremors, muscle rigidity, bradykinesia, and others. Individuals affected by PD suffer postural instability. However, PD is also associated with a range of non-motor manifestations, notably psychological disturbances such as depression, anxiety, and apathy, as well as cognitive decline and, in advanced stages, dementia [1].

The NDDs affect different predominant brain regions: hippocampus (AD), basal ganglia (Parkinson’s and Huntington’s disease), and motor neurons (ALS) [10]. The exact etiology of most NDDs still remains unclear. However, several risk factors are recognized, including genetic mutations (e.g., mutations in *APP*, *PSEN1*, and *HTT* genes), inflammation, and oxidative stress. Nowadays, it is considered that chronic brain inflammation may significantly accelerate neurodegeneration. Aging is also regarded as a risk factor for most NDDs. Environmental exposure to toxins or head injuries are also considered risk factors [1].

NDDs involve the progressive loss of neurons, the building blocks of the nervous system. This loss is often irreversible and occurs through various mechanisms, including protein misfolding, mitochondrial dysfunction, oxidative stress, excitotoxicity, and chronic inflammation. Many NDDs are associated with the accumulation of misfolded or abnormal proteins, such as amyloid-β in Alzheimer’s disease, α-synuclein in Parkinson’s disease, and huntingtin protein in Huntington’s disease. These proteins aggregate in brain tissue, forming plaques or tangles that disrupt neuronal communication and trigger cell death.

At present, NDDs remain incurable. The therapeutic strategies are primarily focused on alleviating symptoms and decelerating disease progression. Pharmacological interventions are frequently employed to manage clinical symptoms—acetylcholinesterase inhibitors are prescribed for AD, while levodopa remains the standard treatment for motor symptoms in PD. A major challenge still remains detecting NDDs in early stage and distinguishing between similar conditions due to overlapping symptoms [11]. Non-pharmacological interventions, including physiotherapy, cognitive rehabilitation, nutritional management, and psychosocial support, are essential for sustaining patients’ functional abilities and overall quality of life [12,13]. Moreover, herbal medicine presents promising strategies for slowing the progression of AD and alleviating its symptoms. The development and commercialization of plant-based medications are rapidly gaining traction, reflecting their growing scientific and economic importance in the healthcare industry. Several plant-derived products have undergone rigorous standardization, with their efficacy and safety validated for specific therapeutic applications. Notable examples include *Ginkgo biloba*, *Bacopa monnieri*, *Salvia officinalis*, *Curcuma longa*, *Rosmarinus officinalis*, *Melissa officinalis*, *Galanthus nivalis*, *Lepidium meyenii*, and *Centella asiatica*. A range of bioactive phytochemicals—including polyphenols, lipophilic vitamins, long-chain omega-3 polyunsaturated fatty acids, isothiocyanates, and carotenoids—exhibit neuroprotective properties and have been implicated in modulating molecular pathways associated with Alzheimer’s disease pathogenesis. These compounds represent promising candidates for the development of prophylactic and therapeutic interventions aimed at mitigating neurodegeneration [12,13].

A range of innovative therapeutic approaches is currently under investigation, such as stem cell-based therapies, gene-editing platforms such as CRISPR, and immunotherapies directed at pathogenic proteins. Moreover, phytochemicals such as β-caryophyllene and xanthohumol have demonstrated significant neuroprotective potential in preclinical models. These natural agents exert multifaceted effects by modulating neuroinflammatory pathways, oxidative stress responses, and aberrant protein aggregation—pathological mechanisms that are central to the development and progression of neurodegenerative disorders.

The aim of this review is to provide a comprehensive synthesis of current scientific findings on the neuroprotective potential of the natural compounds β-caryophyllene and xanthohumol. By examining their biological effects and recent advancements in optimization through nano- and microscale drug-delivery systems, current work offers a better understanding of their therapeutic relevance.

## 2. Results and Discussion

### 2.1. Studies Evaluating β-Caryophyllene Activity on NDDS

**Figure 1:**
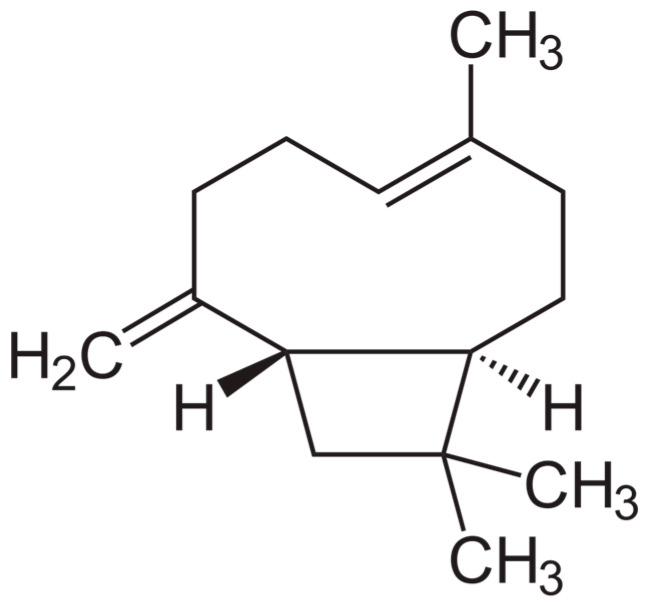
Chemical structure of β-caryophyllene.

β-Caryophyllene (BCP) is a naturally occurring bicyclic sesquiterpene (Figure 1). Several plant species are known to be rich in BCP, including *Syzygium aromaticum*, the rhizome of *Zingiber nimmonii*, *Helichrysum* species, the leaves of *Callistemon linearis*, the aerial parts of *Salvia verticillata*, and the leaves of *Humulus lupulus* and *Stachys lanata*.

The compound is a selective agonist of cannabinoid type 2 receptors (CB2-R). Unlike the compounds that interact with cannabinoid type 1 receptors (CB1), BCP lacks psychoactive effects due to its minimal CB1 affinity. Among its diverse biological roles, BCP demonstrates anti-inflammatory properties by suppressing key inflammatory mediators, including inducible nitric oxide synthase (iNOS), interleukin-1β (IL-1β), interleukin-6 (IL-6), tumor necrosis factor-α (TNF-α), nuclear factor kappa B (NF-κB), and cyclooxygenases COX-1 and COX-2. Furthermore, its actions are partly mediated through activation of peroxisome proliferator-activated receptors, particularly PPAR-α and PPAR-γ.

**Figure 2:**
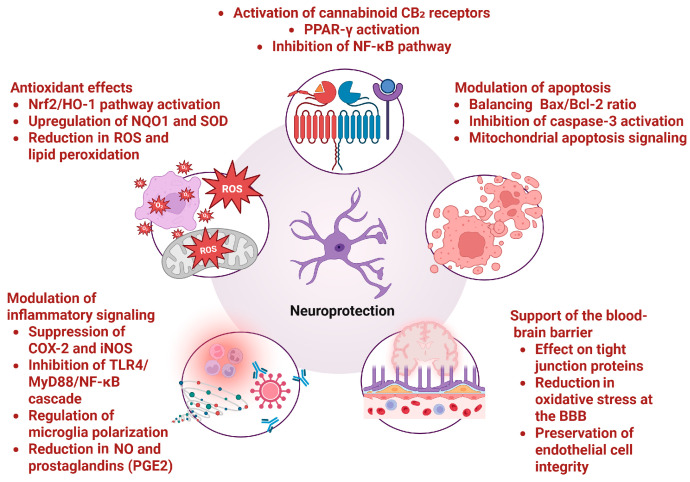
β-Caryophyllene mechanisms of neuroprotection (created with BioRender https://BioRender.com/8ogb0lz, accessed on 6 August 2025)

Based on several in vivo and in vitro studies, it can be concluded that BCP possesses significant neuroprotective activity (Table 1). The possible mechanisms of neuroprotection are presented in Figure 2.

Ojha, Shreesh, et al. demonstrated that the neuroprotective effect of the cannabinoid in a model of PD was mediated by its anti-inflammatory and antioxidant activities [27]. Its antioxidant activity was also established by Flores-Soto, M. E., et al., as the phytocannabinoid enhanced the activity of NAD(P)H quinone oxidoreductase (NQO1) [33]. The anti-inflammatory effect in a transgenic APP/PS1 AD model was confirmed by the activation of the CB2 receptor and PPARγ pathway by BCP [26]. Furthermore, BCP alleviated PD-associated BBB disruption and oxidative stress by reducing the selective death of dopaminergic neurons [36] and also relieved motor dysfunction [28].

Some in vitro studies demonstrated the neuroprotective activity in SH-SY5Y cell lines, as BCP enhanced cell viability, reduced the release of lactic dehydrogenase, decreased the generation of reactive oxygen species (ROS), and altered Caspase 3 activity [17,23]. Gouthamchandra, Kuluvar, et al. proved that standardized extract of BCP from black pepper exerted both neuroprotective and anti-inflammatory activities by suppressing COX-2, iNOS, and mitogen-activated protein kinase (MAPK) signaling pathway [20]. In vitro protective effects of BCP were also established in Aβ1–42-induced neuroinflammation, which is associated with AD [18]. In a model of AD, BCP showed neuronal protection and antagonism of Aβ neurotoxicity by inhibiting the “JAK2-STAT3-BACE1” signaling pathway [21]. In vivo and ex vivo research utilizing models of chronic MS, along with in vitro studies, demonstrated the neuroprotective, anti-inflammatory, and immunomodulatory effects of BCP [24,29,31,34].

Askari, Vahid Reza, and Reza Shafiee-Nick demonstrated that BCP at low doses exerted its effects through CB2 receptors, while at higher concentrations the protective activity decreased and the PPAR-γ pathway was activated [19]. Another study revealed the neuritogenic potential of BCP, suggesting it may provide neuroprotection through a mechanism that does not involve cannabinoid receptors [15].

### 2.2. Studies Evaluating Xanthohumol Activity on NDDS

**Figure 3:**
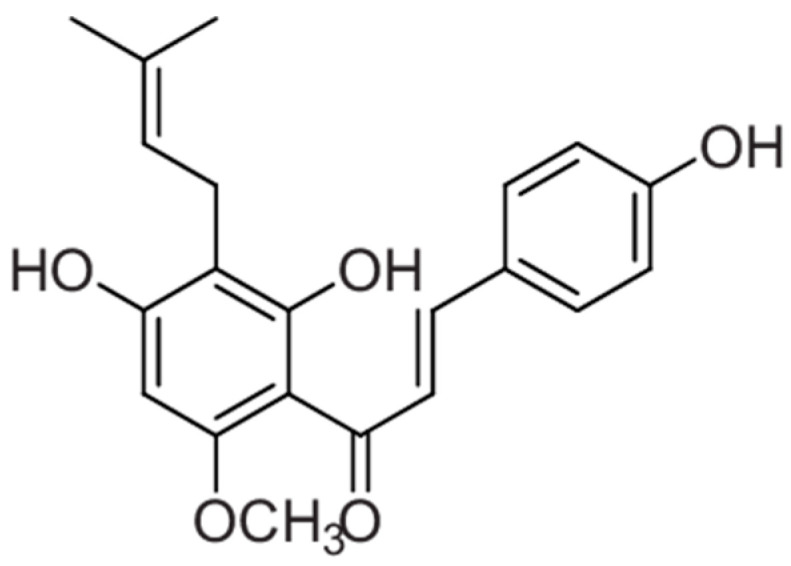
Chemical structure of xanthohumol.

Xanthohumol ((*E*)-1-[2,4-dihydroxy-6-methoxy-3-(3-methylbut-2-enyl)phenyl]-3-(4-hydroxyphenyl)prop-2-en-1-one, XAN, (Figure 3) is a relatively simple prenylated chalcone found in the *Humulus lupulus* L., Cannabaceae (hop plant), where it serves as the main prenylflavonoid in the female flowers, commonly known as hop cones. Hops are primarily used to impart bitterness and flavor to beer, making beer the main dietary source of XAN and similar compounds. In recent years, XAN and other prenylated chalcones have drawn considerable scientific interest for their potential role in cancer prevention. Meanwhile, hop-based herbal products are being sold—often marketed for purposes such as breast enhancement in women—without sufficient testing for safety or effectiveness. Although the market for such herbal products is small and not actively supported by the hop farming industry, growers are exploring new uses for hops and their constituents [38].

XAN has been shown to counteract cognitive impairments caused by a high-fat diet [39].

Hop extracts containing XAN promote sleep and neuroprotection, with enhanced potential for antioxidant and neuroprotective activity [40].

Xanthohumol, a prenylchalcone, shows significantly lower activity in stimulating neuronal differentiation in adult neural stem cells compared to pyranochalcones, suggesting that the pyrano ring is a key structural element. However, XAN has been reported to possess neuroprotective properties [41]. Moreover, XAN may enhance cognitive flexibility in mice [42]. According to Legette et al., XAN bioavailability in rats is dose-dependent, with higher doses resulting in lower relative bioavailability [43].

Xanthohumol has attracted significant attention for its neuroprotective potential in neurodegenerative diseases, particularly AD and PD. In vitro and in vivo studies are presented in Table 2.

**Figure 4:**
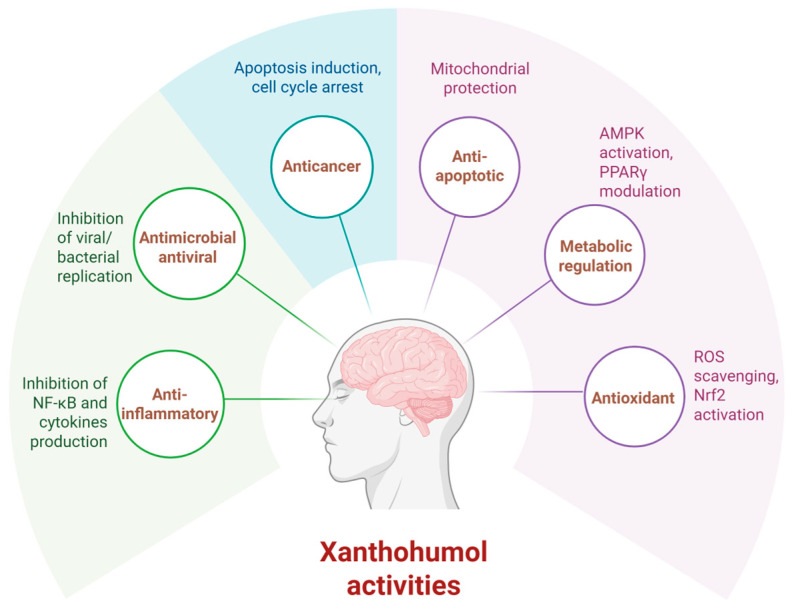
Biological activities of xanthohumol (created with BioRender. https://BioRender.com/v19cxxt, accessed on 6 August 2025).

The findings summarized in the Table 2 provide a comprehensive overview of the multifaceted mechanisms through which XAN exerts its therapeutic effects, including modulation of protein aggregation, antioxidant activity, anti-inflammatory responses, neurotransmitter regulation, and enhancement of neuronal survival (Figure 4).

A key neuroprotective mechanism of XAN involves the inhibition of pathological protein aggregation. XAN has been shown to directly inhibit the fibrillization tau protein and disaggregate existing fibrils, effectively reducing tau-induced apoptosis in cellular models of AD [48]. In parallel, XAN and its derivatives have demonstrated the ability to inhibit the aggregation and fibrillation of amyloid-β (Aβ1-42), forming less-toxic amorphous aggregates and preventing β-sheet formation, which is crucial in mitigating amyloid pathology [52,67].

Oxidative stress and neuroinflammation are pivotal contributors to neurodegeneration, and XAN has been consistently shown to activate the Nrf2/HO-1 pathway, enhancing antioxidant defenses and reducing reactive oxygen species (ROS) levels [45,49,50,56,58,62]. These effects have been observed in models of ischemic stroke, LPS-induced inflammation, corticosterone-induced cytotoxicity, and iron overload. Furthermore, XAN reduced the expression of pro-inflammatory cytokines such as TNF-α and IL-1β, and inhibited signaling pathways such as NF-κB and p38-MAPK, suggesting its efficacy in curbing neuroinflammatory responses [54,55,56,58].

XAN also modulates several key signaling pathways associated with neurodegeneration. For instance, it regulates kinases such as GSK3β and phosphatases such as PP2A, which are involved in tau hyperphosphorylation, and influences endoplasmic reticulum stress and proteasome function [47]. In vivo studies in APP/PS1 transgenic mice revealed that XAN reduced excitotoxicity by decreasing glutamate levels, enhancing mitochondrial function, and promoting mitophagy, thereby supporting synaptic health and memory function [63]. Additionally, XAN was shown to suppress autophagosome maturation via VCP binding in certain contexts, although in others, it promoted autophagy and inhibited apoptosis, highlighting the complexity and context-specific nature of its effects [44,59].

Beyond its antioxidant and anti-apoptotic roles, XAN modulates the adenosinergic system by enhancing A1 receptor expression, which may reduce excitotoxicity—a critical process in AD progression [51,53]. Moreover, its ability to inhibit cholinesterases (AChE and BChE) supports its potential for symptomatic treatment of AD, akin to currently approved cholinergic drugs [46].

Emerging evidence also implicates the gut–brain axis in XAN’s neuroprotective mechanisms. Studies demonstrated that XAN modulates gut microbiota composition and metabolite profiles, thereby contributing to improved cognition and reduced neuroinflammation in AD models [53,55,64]. These findings open new avenues for XAN in systems-level interventions targeting neurodegeneration.

Despite its therapeutic promise, XAN’s clinical translation is hindered by poor oral bioavailability and limited BBB penetration. To address these limitations, several formulation strategies have been developed. Nanostructured lipid carriers, solid dispersions, self-emulsifying drug-delivery systems (SNEDDSs), and cyclodextrin complexes have significantly enhanced XAN’s solubility, stability, and brain bioavailability, resulting in improved cognitive and motor function in animal models [46,62,65,68].

XAN exhibits a broad spectrum of neuroprotective activities relevant to the pathology of neurodegenerative diseases. Its ability to target oxidative stress, inflammation, protein misfolding, synaptic dysfunction, and neurotransmitter imbalance makes it a compelling candidate for future therapeutic development. Continued research, particularly well-designed clinical studies and optimized drug-delivery systems, will be critical to unlocking XAN’s full potential in treating AD, PD, and related disorders.

Despite the promising neuroprotective potential of XAN in the management of PD, its clinical application is hindered by poor solubility, low bioavailability, and limited permeability across the BBB, ultimately resulting in reduced therapeutic efficacy. To address these challenges, a solid dispersion formulation of XAN was developed. This strategy significantly enhanced the delivery of XAN to the brain, leading to increased dopamine levels and reduced oxidative stress and inflammation—critical pathological features implicated in PD progression [69].

Similarly, XAN has demonstrated both neuroprotective and senolytic properties in the context of AD, yet its therapeutic impact remains constrained by the same pharmacokinetic limitations. To overcome these barriers, a self-nanoemulsifying drug-delivery system (SNEDDS) was formulated. In vivo studies demonstrated that XAN-loaded SNEDDS significantly improved cognitive and motor functions while reducing AChE activity, Aβ levels, oxidative stress, and neuroinflammation in rat models of AD [40]. These findings underscore the potential of advanced drug-delivery strategies in enhancing the bioavailability, stability, and therapeutic efficacy of XAN for neurodegenerative diseases.

### 2.3. Drug-Delivery Systems for β-Caryophyllene

**Figure 5:**
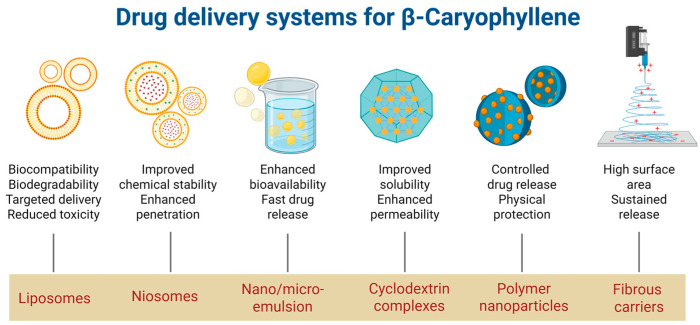
Drug-delivery systems for β-Caryophyllene (created with BioRender. https://BioRender.com/0bywlj6, accessed on 7 August 2025).

The pharmacological potential of β-caryophyllene as a neuroprotective, anti-inflammatory, and analgesic agent has generated considerable interest in developing effective delivery systems to overcome its inherent limitations, including low aqueous solubility, high lipophilicity, chemical instability, and extensive first-pass metabolism. In recent years, a variety of advanced carriers (Table 3), primarily nanotechnology-based, have been designed to improve its bioavailability, stability, and therapeutic efficacy (Figure 5).

Lipid-based nanocarriers are among the most widely investigated, leveraging BCP’s lipophilic nature. Nanostructured lipid carriers combine solid and liquid lipids into a matrix that entraps BCP, resulting in high encapsulation efficiency (87%), particle sizes of 200–250 nm, and sustained release profiles. These nanostructures enhance solubility, protect BCP from volatilization and oxidation, and improve membrane penetration, offering superior pharmacokinetic and pharmacodynamic properties compared to conventional formulations [70]. Similarly, self-emulsifying drug-delivery systems, which spontaneously form fine oil-in-water emulsions upon contact with aqueous media, encapsulate BCP in 40–50 nm droplets, significantly increasing its oral bioavailability, reducing inter-subject variability, and accelerating absorption [71]. Liposomes, particularly those prepared with soy phosphatidylcholine, encapsulate BCP in bilayer vesicles, improving dispersibility and cellular uptake. Studies have shown that BCP-loaded unilamellar and multilamellar liposomes enhance cytotoxic effects on cancer cells compared to free BCP, with stability and release profiles depending on the lipid-to-drug ratio [72]. A notable advancement in this area is Rephyll^®^, a liposomal BCP powder produced via nanofiber weaving technology, which demonstrated high encapsulation efficiency, sustained release, long shelf life, and improved muscle recovery in a clinical study [73]. Another liposomal formulation improved neurological outcomes and preserved BBB integrity in a rat model of subarachnoid hemorrhage, underscoring the neurovascular protective potential of liposomal BCP [74].

Emulsion-based carriers have also been extensively employed for BCP delivery. Both nanoemulsions and microemulsions, which are isotropic oil–surfactant–water mixtures with nanodroplets typically less than 200 nm, improve BCP stability, controlled release, and tissue distribution. Incorporating nanoemulsions into hydrogels enhances their handling and residence time at the target site, resulting in improved penetration and anti-inflammatory activity [75]. On the other hand, microemulsion-based hydrogels have demonstrated even greater entrapment, skin retention, and pharmacological efficacy, attributable to their thermodynamic stability and superior globule characteristics [76]. Microemulsions using copaiba oil-resin as both the oil phase and the active ingredient protected BCP from oxidation and enhanced its antimicrobial and anti-inflammatory activity in vitro and in vivo [77]. A complementary approach involves using BCP itself as a multifunctional oily core in nanoemulsions. In another study, nanoemulsions co-loaded with indomethacin in a BCP-rich core exhibited synergistic anti-inflammatory effects, reduced the required doses, and demonstrated favorable physicochemical and safety profiles, highlighting the multifunctionality of BCP as both an active ingredient and carrier [78]. Furthermore, the choice of lipid plays a critical role in the performance of BCP-loaded carriers. Medium-chain triglyceride carriers exhibited superior stability, digestion, and bioaccessibility compared to those with longer or unsaturated fatty acids [79]. HLB-guided (hydrophilic lipophilic balance) formulation strategies have also been used to optimize nanoemulsion properties, producing stable, monodisperse systems with high encapsulation efficiency and biphasic release [80].

Cyclodextrin-based delivery systems have been explored to enhance the solubility and biological activity of BCP. Complexes of BCP with methyl-β-cyclodextrin (MβCD) increased solubility approximately tenfold, protected it from degradation, and enhanced its anti-inflammatory, antioxidant, and gastric protective effects in vivo [81]. A similar inclusion complex prepared by coprecipitation improved BCP’s solubility, stability, and oral bioavailability by 2.6-fold compared to free BCP [82].

In addition to lipid- and cyclodextrin-based carriers, niosomes have been adapted for BCP delivery. PLGA-modified, pH-sensitive niosomes achieved high encapsulation efficiency, controlled release in acidic environments, and enhanced cytotoxicity against triple-negative breast cancer cells, demonstrating their potential as advanced, responsive delivery systems [83].

Polymeric nanoparticles are another promising approach, offering controlled and targeted delivery. When modified with polyethylene glycol (PEG), nanoparticles stabilize BCP in a hydrophilic matrix, achieving 98% encapsulation and forming uniform particles (<150 nm), with potential for brain targeting [29]. PEG–PLGA nanoparticles (350 nm) enhance BCP stability, oral bioavailability, and pharmacokinetics, as PEG reduces mucoadhesion and extends circulation [84]. Chitosan-based nanoparticles, which are positively charged and bioadhesive, improve BCP activity, decrease toxicity, and increase tissue retention [85].

Beyond conventional carriers, other innovative delivery platforms have been reported. Intranasal nanoemulsions demonstrated efficient brain delivery of BCP in a seizure model, with high encapsulation efficiency, stability, and anticonvulsant effects [86]. Solid fibrous carriers, such as edible hemp, coconut, or rice fibers, provided rapid mucosal absorption and sustained oral release of BCP in a patented formulation (US10933016B2). Inhalation-based systems using handheld vaporizers enabled controlled pulmonary delivery of pure BCP (CN110225748A/WO2018094359A1), while clinically formulated products have been patented for schizophrenia (EP2827846A1), pain (EP4364730A1), multifunctional phospholipid–triglyceride systems (US11202765B2), and mild cognitive impairment (US11911346B2), illustrating the wide therapeutic potential of optimized BCP delivery strategies.

Apart from its role as an active compound, BCP has been investigated as a natural penetration enhancer. Studies have demonstrated that BCP fluidizes and disrupts stratum corneum lipids, increasing the permeability of hydrophilic drugs with minimal irritation [87]. Spectroscopic evidence confirmed its incorporation into bilayers, disturbing hydrophobic and subpolar regions, particularly in cholesterol-rich membranes, thereby increasing permeability [88]. Finally, a green, catalyst-free process for oxidizing BCP into caryophyllene oxide, described in patent WO2020069754A1, yields stable epoxide isomers suitable for pharmaceutical and cosmetic use, aligning with sustainable production approaches.

**Table 3:** Drug-delivery systems for β-Caryophyllene, where ↑—increase and ↓—decrease.

Carrier Type	Polymer/Lipid	Impact on BCP Characteristics	Ref.
Nanostructured lipid carriers	Compritol 888ATO and linseed oil	↑ Solubility, ↑ stability, and cumulative release	[70]
Lipid nanocarriers	Medium-chain triglyceride, coconut oil, cocoa butter, olive oil, soybean oil	↑ Stability, ↑ bioaccessibility	[79]
Self-emulsifying drug-delivery system (VESIsorb^®^)	Medium-chain triglycerides, natural vegetable oils, PEG	↑ Oral bioavailability, ↓ inter-individual variability, and fast absorption	[71]
Liposomes	Soybean phosphatidylcholine	↑ Dispersibility, ↑ cellular uptake,	[72]
	Phospholipids	Neuroprotection, BBB repair, ↓brain edema	[74]
Liposomal powder (Rephyll^®^)	Phospholipids	↑ Stability, sustained release, ↑ clinical efficacy	[73]
Hydrogel containing nanoemulsified BCP	Hydroxyethyl cellulose	↑ Stability, controlled release, ↑anti-inflammatory activity	[75]
Microemulsion hydrogel	Isopropyl myristate, Phospholipon 90, Carbopol 940	↑ Skin permeation, ↑ anti-inflammatory activity	[76]
Microemulsion	Copaiba oil-resin	↑ Antimicrobial and anti-inflammatory activity	[77]
Nanoemulsion	Medium chain triglycerides (capric and caprylic acids)	Dose reduction, ↑ anti-inflammatory effect	[78]
	Lecithin, oleylamine	Direct brain delivery, ↑ anticonvulsant effect	[86]
Cyclodextrin inclusion complexes	Methyl-β-cyclodextrin β-cyclodextrin	↑ Solubility, ↑ stability, ↑ oral bioavailability	[81,82]
PEGylated nanoparticles	PEG 400, polyvinyl alcohol (PVA), poly-caprolactone (PCL), β-CD, and chitosan	Controlled release, ↑ stability, ↑ ability to permeate BBB	[89]
Nanoparticles	PEG, PLGA	↑ Stability, prolonged circulation, ↑ oral bioavailability	[84]
	Chitosan	↑ Mucoadhesion and retention, ↓ toxicity	[85]
Fibrous carriers	Natural fibers (hemp, coconut, rice)	↑ mucosal absorption, controlled release,	US10933016B2

### 2.4. Drug-Delivery Systems for Xanthohumol

Due to its poor biopharmaceutical characteristics, mainly its low solubility in water, XAN exhibits insufficient oral resorption due to the restricted dissolution in body fluids [90]. Apart from this, it is also characterized by relatively short half-life and extensive liver biotransformation via processes such as oxidation, reduction, glucuronidation etc. This leads to the formation of multiple metabolites, some of which are bioactive, such as isoxanthohumol, while others do not contain the biological activity of the initial compound [43].

In general, enhancing the solubility of XAN is one of the most commonly used methods for improving its biopharmaceutical characteristics, namely the incorporation of XAN in a complex with cyclodextrins. They are cyclic oligosaccharides with a hydrophobic interior cavity and hydrophilic exterior. They are well-established carriers for enhancing the solubility of hydrophobic drugs and that of natural compounds [91]. In a recent study, Kirchinger et al. developed a xanthohumol/2-hydroxypropyl-β-cyclodextrin complex and reported that, as the concentration of cyclodextrin was increased, a 650-fold increase in the solubility of XAN was observed. They also stated that the in vitro bioactivity of free xanthohumol and that of its complexed form were not significantly different, which showed that xanthohumol was released from the complex unchanged. Furthermore, the scientists conducted an in vivo pharmacokinetics test, which showed that XAN can be detected in the brain and the cerebrospinal fluid for up to 6 h post i.p. administration in mouse models [92].

**Figure 6:**
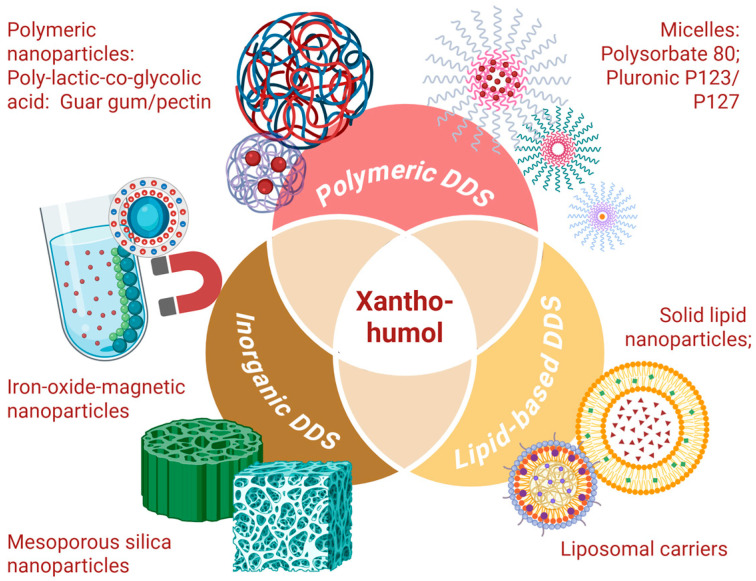
Drug-delivery systems for xanthohumol (created with BioRender. https://BioRender.com/7sxkqpq, accessed on 7 August 2025).

Another popular method for overcoming the poor biopharmaceutical characteristics of XAN is its incorporation in different nanoparticulate systems. They present a great opportunity to increase the solubility, bioavailability and stability of XAN, as well as alter its pharmacokinetics [93]. Over the years, multiple nano-sized drug-delivery systems (DDS), with incorporated XAN, have been developed, which can be classified as polymer-based drug-delivery systems, lipid-based DDS or inorganic-based DDS (Figure 6).

#### 2.4.1. Polymeric DDS for Xanthohumol

Polymeric nanoparticulate systems have been broadly studied as carriers of XAN. The most common technique used is the micellar incorporation of XAN. In a recent study, Khayyal et al. developed micelles consisting of polysorbate 80, with incorporated XAN. The resulting micelles were compared in vivo for their anti-inflammatory effects with pure diclofenac sodium and non-micellized XAN. They reported a significantly higher anti-inflammatory effect of the micellar XAN compared to the native form, which was found not only to reduce the paw volume in arthritic rats but also to decrease the serum levels of cytokines TNF-α and IL-6. The effect was not significantly different from the effect of pure diclofenac sodium, which shows that the incorporation of xanthohumol in micelles significantly increases its efficacy due to the enhancement of its biopharmaceutical characteristics [94]. In another study, Ronka et al. developed Pluronic P123- and Pluronic F127-based micelles loaded with XAN and its primary metabolite, isoxanthohumol. They managed to synthesize micelles with a size of around 30 nm and extremely high encapsulation efficiency varying between 93.5% and 100%. However, the in vitro cytotoxicity assay and in vitro dissolution test showed relatively similar results between micellar-xanthohumol/isoxanthohumol and their native counterparts [95].

Apart from micellar incorporation, several other polymer-based drug-delivery systems have been developed as potential carriers of XAN. Poly-lactic-co-glycolic acid has been broadly utilized as a carrier for several classes of drugs due to its versatility, biocompatibility, and biodegradability [96]. Fonseca et al. (2021) developed xanthohumol-loaded PLGA nanoparticles as a new approach in the treatment of cutaneous melanoma [97]. The synthesized PLGA nanoparticles showed a relatively small average size of around 310 nm and loading efficiency of up to 90%. The developed structures showed sufficiently higher cytotoxic effects against malignant cutaneous cell-lines, compared to non-encapsulated XAN. This makes PLGA-based nanoparticles a suitable carrier for XAN, significantly increasing its therapeutic potential [97].

Similar nano-sized structures have been developed by Ghosh et al.; however, they were developed as a tool for the protection of the corneal epithelial cells. They reported that the formulated structures had a cytoprotective effect in in vitro oxidative stress injury in human corneal epithelial cells and significantly improved dry eye disease symptoms in a mouse model [98].

A polysaccharide-based drug-delivery system was also used as a potential carrier of xanthohumol. Hanmantrao et al. developed a guar gum/pectin based self-nanoemulsifying system for the colon-targeted drug-delivery of xanthohumol. The developed formulation showed increased xanthohumol solubility in water due to the transformation of its structure from crystalline into amorphous form. In addition, the formulation showed a 1.5-fold increase in the cytotoxic effect of XAN against Caco-2 cells, compared to free xanthohumol [99].

All the above-mentioned studies underscore the potency of polymer-based drug-delivery systems for XAN, enhancing its biopharmaceutical characteristics and its therapeutic potential.

#### 2.4.2. Lipid-Based DDS for Xanthohumol

Apart from polymeric systems, different lipid-based formulations, such as liposomes and solid lipid nanoparticles, have also been reported as potential carriers of XAN. Solid lipid nanoparticles were found to be extensively utilized due to their advantages, including high colloidal stability, biocompatibility, and non-toxicity. Harish et al. developed xanthohumol-loaded solid lipid nanoparticles, which were reported to be in the 100 nm-range in terms of their size and high entrapment efficiency of XAN, around 80%. DSC (differential scanning calorimetry) and PXRD (powder x-ray diffraction) analyses showed that the xanthohumol structure shifted from a crystalline to an amorphous form; however, the release of xanthohumol was significantly slow; at 92 h, only 28% of the drug was released, which was probably due to the immobilization of xanthohumol in the lipid matrix. Although the drug release was slow, the formulated xanthohumol-loaded structures showed enhanced pharmacokinetic properties compared to non-encapsulated XAN [100].

In a recent study, Khandale et al. successfully addressed the issue of slow drug release by developing a novel solid lipid nanoparticle structure which showed a 5-fold increase in the in vitro drug release rate, compared to native xanthohumol. The formulation was tested in vivo in AlCl_3_-induced AD mouse models. The data showed no statistically significant differences between the animal groups treated with solid lipid nanoparticles loaded with xanthohumol and the standard treatment with donepezil. The pharmacokinetic study showed an 11.3-fold increase in the brain concentration of xanthohumol after incorporation in solid lipid nanoparticles, compared to the non-encapsulated xanthohumol, which shows the significance of the nano-sized drug-delivery systems in the transportation of molecules across the BBB [66].

Liposomal carriers of xanthohumol have also been developed previously. Buczek et al. investigated the effect of cyclodextrins on characteristics of xanthohumol-loaded liposomes. They reported that the complexation of xanthohumol with cyclodextrin modulates the temperature-dependent release of XAN from the synthesized structures [101].

#### 2.4.3. Inorganic Carriers for Xanthohumol

While less studied compared to polymeric and lipidic DDSs, inorganic carriers receive great attention from the scientific community due to their unique optical and magnetic properties. They can be utilized for targeted drug delivery and for diagnostic purposes, which makes them highly versatile carriers of different active substances [102].

Iron oxide magnetic nanoparticles are one of the most extensively researched inorganic carriers for magnetically assisted drug delivery. Matthews et al. developed xanthohumol-loaded iron oxide nanoparticles coated with polyethylene glycol-block-allyl glycidyl ether, as a potential tool for the treatment of multiple myeloma. The prepared structures showed a high loading efficiency of 80%. The in vitro dissolution assay showed that <5% of xanthohumol was released after 48 h, whereas after stimulation for 15 min, > 40% of the incorporated xanthohumol was released. The developed structures showed high cytotoxic effects against multiple myeloma cells, which was additionally amplified by the generation of reactive oxygen species from the iron carriers [103].

Mesoporous silica nanoparticles are another attractive drug-delivery tool, possessing numerous advantages due to their porous structure and relatively high surface area, biocompatibility, and non-toxicity. Their pore-size is easily controlled and allows extensive surface modification, providing stimuli-responsive behavior, control of the drug release process, targeted delivery of genes, and diagnostic capabilities [104].

Krajnovic et al. developed XAN-loaded mesoporous silica nanoparticles and studied their cytotoxic effects against melanoma cells. They reported a relatively low entrapment efficiency of XAN, ranging between 8 and 17%. They tested the formulation in vitro for its antitumor activity. It was determined that the cell-death effect induced by the xanthohumol-loaded mesoporous particles involves inhibition of cell proliferation and an autophagic cell death mechanism, whereas free XAN induces apoptosis. This shows that the incorporation of xanthohumol in mesoporous silica nanoparticles can alter the antitumor effect of xanthohumol qualitatively as well as quantitatively [105].

### 2.5. Clinical Trials and Future Perspectives

Despite their promising biological effects, both BCP and xanthohumol are not yet FDA-approved pharmaceuticals, which may be related to some of their poor biopharmaceutical characteristics [93,106]. However, they remain subjects of significant scientific interest. A thorough search through of the database clinicaltrials.gov [107] shows that there are quite a few clinical trials for both compounds, which are presented in Table 4. Although none of the studies are directly related to neurodegenerative diseases, most of them aim to evaluate the potential of the molecules as antioxidants and anti-inflammatory drugs. Some of the trials aim to determine the pharmacokinetics of XAN and BCP, which can provide a better understanding of their biopharmaceutical behavior.

Although β-caryophyllene and xanthohumol are generally considered safe and are available as dietary supplements, their long-term safety profiles in patients with neurodegenerative diseases are not well characterized. Preclinical and early clinical data suggest that these compounds have low toxicity; however, they both interact with important metabolic pathways. β-Caryophyllene acts as a CB_2_ receptor agonist and a PPAR-γ activator, which may influence immune responses and potentially interact with other anti-inflammatory or immunomodulatory medications. On the other hand, xanthohumol undergoes extensive metabolism in the liver, such as glucuronidation and oxidation, which raises concerns about interactions with cytochrome P450 substrates or drugs that have narrow therapeutic windows. Additionally, the possibility of additive effects with conventional antioxidants or cholinesterase inhibitors cannot be ruled out. To ensure safe clinical use, it is crucial to conduct thorough safety assessments, pharmacokinetic–pharmacodynamic studies, and a systematic evaluation of potential drug–drug interaction risks.

Regarding neuroprotection, research on β-caryophyllene and xanthohumol is still predominantly at the in vitro or in vivo animal study stage. To translate these findings into clinical practice, well-designed human trials are essential to confirm safety, efficacy, and optimal dosing. Achieving regulatory approval will require not only robust clinical evidence but also advanced formulation strategies to overcome bioavailability limitations. Polymer- and lipid-based nano/microsystems, micellar carriers, and self-emulsifying drug-delivery systems offer promising solutions. Future work should also explore biomarker-driven studies and potential synergistic effects in combination therapies. By integrating formulation science, clinical pharmacology, and neuroscience, the therapeutic potential of β-caryophyllene and xanthohumol could be fully realized, paving the way for novel strategies in neurodegenerative disease management.

## 3. Materials and Methods

The literature search aimed to identify studies investigating β-caryophyllene and xanthohumol as neuroprotective agents. The search strategy employed a combination of relevant keywords, including β-caryophyllene, xanthohumol, neuroprotective agents, neuroprotection, Parkinson’s disease, Alzheimer’s disease, structure–activity relationship, drug-delivery systems, animal studies, cell culture studies and clinical trials. Only studies that specifically addressed NDDs and DDSs in relation to these two phytochemicals were considered eligible for inclusion. After the initial screening and removal of duplicates, the selected articles were read in full to confirm their relevance.

## 4. Conclusions

Neurodegenerative diseases currently have no cure, but β-caryophyllene and xanthohumol show promise as natural therapeutic agents. Preclinical studies suggest BCP may effectively target Parkinson’s disease and multiple sclerosis by activating the CB2 receptor and PPAR-γ, which modulate neuroinflammation, oxidative stress, and blood–brain barrier integrity. It also reduces amyloid-β neurotoxicity and tau aggregation, although evidence for Alzheimer’s disease is less comprehensive. Conversely, XAN consistently demonstrates efficacy in Alzheimer’s models by inhibiting amyloid-β and tau aggregation, reducing excitotoxicity, activating the Nrf2/HO-1 pathway, and modulating cholinesterase activity. While both compounds share neuroprotective mechanisms, such as anti-inflammatory signaling and antioxidant defense, BCP primarily targets immune pathways, whereas XAN focuses on protein aggregation and redox balance, guiding disease-focused development. At present, BCP and XAN cannot yet be classified as established therapeutic drugs. Rather, they should be regarded as bioactive natural modulators with pronounced neuroprotective properties, holding promise as lead structures or precursors for rational drug design. While no pharmaceutical drug products based on these molecules have yet been registered, β-caryophyllene and xanthohumol are already available as dietary supplements, and the interest in them is steadily growing. This is further supported by the numerous studies in recent years aiming to confirm their efficacy and to develop suitable drug formulations that can enhance their pharmacokinetic characteristics. In this regard, polymer- and lipid-based nano- and microscale drug-delivery systems have emerged as particularly promising carriers for β-caryophyllene and xanthohumol. It is probably only a matter of time before these formulations successfully pass clinical trials and become effective therapeutic strategies for the treatment of neurodegenerative diseases.

## Figures and Tables

**Table 1:** Studies presenting the pharmacological potential of β-caryophyllene, with a focus on its neuroprotective activity.

Study Objectives	Study Design	Main Results	Mechanism of Action	Ref.
To evaluate the protective effects of BCP against glutamate-induced cytotoxicity in C6 glioma cells.	Cells: C6 rat glioma cells (ATCC CCL-107). Treatments: Glu-induced cytotoxicity: Cells exposed to 0.05–10 mM Glu for 24 h. BCP co-administration: Glu (1 mM) + BCP (0.5–1 µM) for 1 or 24 h. BCP pretreatment: BCP (0.5–3 µM) for 24 h before Glu (1 mM) exposure.	BCP inhibited ROS production and reestablished the mitochondrial membrane potential (CB2R/Nrf2 signaling pathway), which led to the prevention of C6 glioma cell line from Glu-induced cytotoxicity.	BCP activated Nrf2 and improved antioxidant defenses, partly via CB2 receptor activation.	[14]
To investigate whether BCP promotes neuritogenesis independently of CB2 receptor activation.	Cell lines: PC12 and SH-SY5Y. Treatment: PC12 cells treated with BCP (10–50 μM) ± NGF or K252a (TrkA inhibitor) for 72 h; SH-SY5Y cells treated with BCP (10 μM) ± retinoic acid for 72 h.	BCP stimulated the process of neuritogenesis and synaptogenesis in PC12 cells through a mechanism that did not involve CB2 receptors.	Activation of NGF-specific receptor trkA. Neuritogenesis and axonal protein upregulation, independent of CB2 or NGF expression.	[15]
To investigate whether trans-caryophyllene (TC) protects against Aβ1-42-induced neuroinflammation in microglial cells, relevant to AD.	BV-2 microglial cells were pretreated with TC (10, 25, 50 μM) and then stimulated with Aβ1-42. Cytotoxicity, NO/PGE2 production, iNOS/COX-2 expression, pro-inflammatory cytokines, TLR4 expression, and NF-κB signaling were assessed.	Pretreatment of BV2 microglial cells with BCP before Aβ stimulation resulted in inhibition of NO, prostaglandin E2 (PGE2) production, inducible nitric oxide synthase (iNOS) and cyclooxygenase-2 (COX-2) expression, as well as secretion of proinflammatory cytokines. Activated overexpression of toll-like receptor 4 (TLR4), phosphorylation and degradation of IκBα, nuclear translocation of p65, and transcriptional activity of NF-κB, induced by Aβ1-42 were reduced.	-	[16]
To evaluate the neuroprotective effects of BCP against MPP+-induced neurotoxicity in SH-SY5Y cells.	SH-SY5Y cells were treated with MPP+ (50 μM) ± BCP (1 or 2.5 μM) for 24 h.	BCP enhanced cell viability, reduced the release of lactic dehydrogenase, and decreased the generation of reactive oxygen species (ROS).	BCP protects via CB2R activation, reducing oxidative stress, preventing apoptosis, and modulating HO-1/JNK pathways.	[17]
To investigate the protective effects of BCP against LPS-induced oligodendrocyte toxicity and the involvement of CB2, Nrf2, PPAR-γ, and SMase pathways.	OLN-93 oligodendrocyte cells treated with LPS; BCP tested at low (0.2–1 μM) and high (10–50 μM) concentrations; effects on ROS, NO, TNF-α, GSH, and signaling pathways were measured; SMase inhibitors (imipramine, fluoxetine) tested for synergy.	The protective effects of BCP are mediated through the CB2 receptor in different pathways at high and low concentrations. BCP prevented the increased production of nitric oxide (NO), ROS, and tumor necrosis factor (TNF-α).	BCP protects oligodendrocytes via CB2 activation, modulating Nrf2/HO-1 (low dose) and PPAR-γ (high dose), with additional benefit from SMase inhibition.	[18]
To evaluate the protective effects of BCP, a selective CB2 agonist, on LPS-induced microglial inflammation and M1/M2 imbalance and to identify signaling pathways involved (CB2, PPAR-γ, SMase).	Cells: Primary microglia isolated from adult C57BL/6 mice. Treatments: BCP dose-response: 0.2–25 µM or JWH-133 (1 µM) ± LPS (1 µg/mL) for 24 h. CB2 involvement: Pre-incubation with AM630 (1 µM) 30 min before BCP/JWH-133 + LPS.	A low concentration of BCP showed selective anti-inflammatory activity. BCP modulated microglia and could affect neuroinflammatory conditions and microglial cells.	-	[19]
To evaluate the anti-inflammatory and neuroprotective effects of Viphyllin, a standardized BCP extract, in macrophages and neuronal cells under oxidative stress.	Cells: RAW 264.7 (macrophages), SH-SY5Y (neuroblastoma). Viability: RAW 264.7: 5–50 μg/mL Viphyllin, 24 h; SH-SY5Y: 12–24 μg/mL Viphyllin ± H_2_O_2_ (500 μM). NO Production: RAW 264.7: 5–50 μg/mL Viphyllin + LPS (1 μg/mL), 24 h.	Regulated LPS-mediated inflammation in macrophages and exerted an antiapoptotic effect against neuronal damage induced by H_2_O_2_.	Anti-inflammatory effects via MAPK pathway inhibition and neuroprotection through anti-apoptotic modulation under oxidative stress.	[20]
To investigate whether BCP exerts neuroprotection in an AD cell model.	PC-12 cells overexpressing amyloid-β precursor protein. Groups: control, empty vector, APP overexpression, and BCP (5, 10, 20 μM).	BCP enhanced the viability of PC-12 cells while protecting cell morphology and counteracted the neurotoxic effects of amyloid-β.	BCP protects neurons by inhibiting the JAK2-STAT3-BACE1 signaling pathway, reducing Aβ-induced toxicity.	[21]
To evaluate whether α-asarone and BCP can inhibit tau fibrillation, disassemble tau fibrils, and protect neuronal cells against tau-induced toxicity.	Tau aggregation and fibrillation were assessed using SDS-PAGE, AFM, ThT/ANS fluorescence, and β-sheet content analysis; SH-SY5Y cells were exposed to 5 μM tau samples treated with α-asarone or BCP for 24 h.	BCP inhibited tau fibrillation and aggregation, leading to the formation of various structural and morphological intermediate species. BCP enhanced cell viability.	-	[22]
To investigate the neuroprotective effects of BCP against rotenone-induced neurotoxicity in SH-SY5Y cells.	In silico: molecular docking of BCP with GSK-3β, NRF2, HO-1. In vitro: SH-SY5Y cells pretreated with rotenone. BCP (100 µg/mL) tested for effects.	BCP increased cell viability, reduced ROS levels, and altered cellular pathways associated with inflammation, redox processes, and apoptosis.	BCP protects cells by modulating the GSK-3β/NRF2/HO-1 axis, reducing oxidative stress, inflammation, and apoptosis.	[23]
To evaluate the immunomodulatory and therapeutic effects of BCP in vitro and in vivo in a mouse model of multiple sclerosis.	Splenocytes from MS-induced C57BL/6 mice treated with BCP (4, 20, 40 μM) were assessed in vitro. In vivo, MS-mice received oral BCP (25 or 50 mg/kg/day).	In vitro and in vivo BCP inhibited the production of NO, H2O2, TNF- α, and Interferon-gamma (IFN-γ). BCP administered orally at a dose of 50 mg/kg/day showed a reduction in the number of inflammatory infiltrates and attenuated neurological damage in the CNS.	-	[24]
To evaluate the anxiolytic, antioxidant, and toxicity effects of BCP in vitro and in vivo.	Swiss albino mice were tested for anxiolytic activity using elevated plus-maze, rota-rod, light/dark, and hiding sphere models. BCP—10, 25, and 50 mg/kg, intraperitoneally. Antioxidant activity was assessed via DPPH, ABTS, and *S. cerevisiae* assays.	BCP exerted dose-dependent anxiolytic and antioxidant effects on experimental animals. It has not shown toxicity in *A. salina*. BCP showed protective and restorative activity in *S. cerevisiae* strains against the harmful effects of hydrogen peroxide.	-	[25]
To evaluate whether BCP prevents cognitive decline and neuroinflammation in an APP/PS1 Alzheimer’s disease model and to determine the roles of CB2 and PPARγ pathways.	APP/PS1 transgenic mice received oral BCP (BCP—16, 48, or 144 mg/kg, orally, 10 weeks). CB2 (AM630) and PPARγ (GW9662) antagonists were used to confirm pathway involvement.	BCP exhibited anti-inflammatory activity, related to the activation of CB2 and PPARγ receptors, which resulted in enhanced memory and cognitive function, a decrease in β-amyloid accumulation, and the suppression of gliosis, as well as the release of pro-inflammatory cytokines.	BCP exerts effects through CB2 receptor activation and subsequent engagement of the PPARγ pathway.	[26]
To evaluate the neuroprotective effect of BCP against rotenone-induced oxidative stress and neuroinflammation in a rat model of PD.	Male Wistar rats. BCP administered once daily for 4 weeks at 50 mg/kg body weight prior to rotenone challenge (2.5 mg/kg body weight).	BCP restored dopaminergic neurons, reduced the levels of proinflammatory cytokines and inflammatory mediators, restored antioxidant enzymes, and prevented lipid peroxidation and the depletion of glutathione.	-	[27]
To investigate the neuroprotective effects and mechanisms of BCP in an MPTP-induced murine model of PD.	PD induce mice model: BCP 10 mg/kg, 5 days (i.p. or oral).	BCP improved motor function, protected dopaminergic neurons, reduced glial activation, and lowered inflammatory cytokines via CB2R.	-	[28]
To investigate the therapeutic effects of BCP on experimental autoimmune encephalomyelitis, a murine model of multiple sclerosis, and to explore the underlying immunomodulatory mechanisms.	C57BL/6 mice were treated with BCP (25 or 50 mg/kg, twice a day, orally).	The immunomodulatory effect of BCP is associated with the inhibition of microglial cells, CD4+ T-lymphocytes, CD8+ T-lymphocytes, and protein expression of pro-inflammatory cytokines. BCP reduced axonal demyelination and modulated the Th1/Treg immune balance.	Neuroprotective and immunomodulatory effects are mediated via CB2 receptor activation.	[29]
To evaluate the effects of a β-caryophyllene–hydroxypropyl-β-cyclodextrin (HPβCD/BCP) inclusion complex on cognitive deficits in a vascular dementia rat model and to investigate underlying mechanisms.	VD rats were treated i.p. for 4 weeks with HPβCD/BCP (16–144 mg/kg), AM630 (3 mg/kg), or saline.	HPβCD/BCP reduced cognitive impairment and nerve fiber loss, enhanced cerebral blood flow, and inhibited neuronal cell death in rats.	BCP demonstrated its neuroprotective properties by stimulating the CB2 pathway.	[30]
To assess the effects of BCP on IL-17, transcription factors (T-bet, GATA-3), and remyelination in mice as a model of MS.	EAE was induced in C57BL/6 mice and treated with BCP (25 or 50 mg/kg/day) by gavage from day 10 post-induction. CNS tissue was analyzed after 9 days of treatment for cytokines, transcription factors, and remyelination.	BCP administered at a daily dose of 50 mg/kg resulted in reduced levels of IL-17 in the brain, medulla, and cerebellum; a reduction in T-bet was observed in both the medulla and cerebellum, while GATA-3 levels increased in the cerebellum.	Anti-inflammatory and neuroprotective effects via modulation of Th1/Th17 (T-bet/IL-17) and Th2 (GATA-3) responses.	[31]
To evaluate the neuroprotective effects of BCP against dementia induced by neuroinflammation and aging in animal models.	Male SD rats: AlCl_3_-induced dementia. Female SD rats: Doxorubicin-induced chemobrain model. D-galactose-induced mitochondrial dysfunction model. BCP given orally at 50 and 100 mg/kg.	At a dosage of 100 mg/kg, BCP had a protective activity against dementia caused by neuroinflammation but did not influence neuronal aging related to mitochondrial dysfunction.	-	[32]
To evaluate the neuroprotective effect of BCP against MPTP-induced Parkinsonism.	Male C57BL/6J mice; 4 groups: 1. saline control, 2. MPTP (5 mg/kg i.p. × 7 days), 3. BCP (10 mg/kg p.o. × 7 days), 4. MPTP + BCP (BCP from day 4 onward, 7 days).	BCP inhibited oxidative stress-induced cell death of dopaminergic neurons and simultaneously increased the expression and enzymatic activity of NQO1.	-	[33]
To evaluate the protective effects of low-dose BCP in an EAE mouse model of MS and explore its CB2-dependent immunomodulatory mechanisms.	Female C57BL/6 mice were immunized with MOG_35–55_ + CFA and pertussis toxin to induce EAE. Mice received low-dose BCP (2.5 or 5 mg/kg/day) ± CB2 antagonist (AM630).	Low doses of BCP influenced EAE, which is a chronic model for MS. The protective impacts of BCP are facilitated by the CB2 receptor. BCP modulated the adaptive (lymphocytes) and innate (microglia) immune systems from an inflammatory (Th1/Th17/M1) to an anti-inflammatory (Th2/Treg/M2) phase.	Protective effects were CB2 receptor–dependent.	[34]
To evaluate the neuroprotective effects of BCP on dopaminergic neurons.	Male C57BL/6 mice were randomly assigned to 4 groups: Sham, 6-OHDA, BCP (10 mg/kg, oral, 5 days), and 6-OHDA + BCP. BCP was administered orally either alone or after 6-OHDA.	Treatment with BCP improved motor dysfunction; exhibited a neuroprotective effect on dopaminergic neurons; and decreased expression levels of NLRP3, caspase-1, and malondialdehyde (MDA).	BCP protects dopaminergic neurons by inhibiting NLRP3 inflammasome activation and reducing oxidative stress.	[35]
To test whether CB2 receptor agonism via BCP can correct blood–brain barrier (BBB) permeability in the MPTP mouse model of PD.	MPTP-induced PD mouse model (male C57BL/6J); 4 groups *n* = 20/group, one of the groups—10 mg/kg, i.g. BCP.	BCP reduced the permeability of the BBB, likely by altering the expression of TJ proteins and decreasing oxidative stress levels.	BCP activation of CB2 reduces BBB disruption and oxidative stress, protecting dopaminergic neurons.	[36]
To determine the effect of BCP supplementation on cognitive function and quality of life in older adults with memory complaints.	Prospective, randomized 8-week trial; 52 participants (mean age 67 ± 5 years, classified obese by BMI); randomized to receive BCP 90 mg (*n* = 29) or 180 mg (*n* = 29). Cognitive performance (4 online brain games) and quality of life assessed at baseline, week 4, and week 8.	BCP is suggested to improve cognitive function in an elderly population.	–	[37]

**Table 2:** In vitro and in vivo studies on the neuroprotective potential of xanthohumol.

Study Objectives	Study Design	Main Results	Mechanism of Action	Ref.
Investigate how XAN affects autophagy at the molecular level.	Human epidermoid carcinoma A431 cells and Hella cells. XAN treatment with 0, 10, 30 μM.	XAN inhibited autophagosome maturation.	XAN binds directly to the N domain of VCP.	[44]
Assess neuroprotective effects of XAN under oxidative stress.	PC12 cells were pretreated with XAN 0.1–0.5 µM.	XAN upregulated cytoprotective genes and protected cells from oxidative damage.	Activated Nrf2 pathway via α, β-unsaturated ketone, enhancing antioxidant defenses.	[45]
Investigate XAN and related hop-derived compounds for their inhibitory effects on AChE and BChE—key enzymes in AD.	In vitro ELISA assays to assess enzyme inhibition and molecular docking for binding interactions of XAN (30–70 µM).	XAN inhibits cholinesterases by binding to active site residues, supporting its potential as a lead compound for AD drugs.	Xanthohumol and 3-hydroxy-xanthohumol showed moderate AChE and BChE inhibition (IC_50_ ~30–70 µM). 8-prenylnaringenin also inhibited BChE.	[46]
Evaluate the therapeutic potential of XAN in AD.	N2a/APP and HEK293/tau cell lines. XAN treatment 52 μM.	XAN reduced Aβ accumulation, inhibited APP processing, and ameliorated tau hyperphosphorylation.	Modulation of PP2A, GSK3β, ER stress, oxidative stress, proteasome function, and cytoskeletal proteins.	[47]
Investigate the effects of XAN on tau protein aggregation related to AD.	Biochemical binding assays, tau aggregation/disaggregation studies, and cell-based toxicity/apoptosis assays. N2a cells were treated with XAN (0–100 μM).	Inhibits aggregation and disaggregates tau fibrils. Reduces tau-induced apoptosis, with low cytotoxicity.	XAN inhibits tau protein aggregation and disaggregate tau filaments.	[48]
Identify XAN analogues that activate Nrf2 and protect against oxidative stress in neurodegeneration.	PC12 cell model, H_2_O_2_/6-OHDA-induced injury, cytotoxicity and Nrf2 pathway analysis. XAN treatment 5 and 10 μM.	Two analogues with removed prenyl group showed low toxicity and rescued cells from oxidative injury.	Activation of Nrf2 pathway via nuclear translocation and protection abolished by Nrf2 knockdown.	[49]
Investigate neuroprotective effects of XAN and quercetin against corticosterone-induced cytotoxicity in cortical cells.	Primary cortical cultures from postnatal day 1 Sprague Dawley male rats were prepared. Quercetin (0.03–3 µM) and XAN (0.2–5 µM) were added for 24 h, then replaced with 200 µM corticosterone for 96 h.	Both polyphenols prevented corticosterone-induced loss of cell viability and restored Bdnf mRNA levels.	XAN neuroprotection is mediated via Nrf2 activation.	[50]
Assess hops and XAN effects on oxidative stress and adenosine receptors.	C6 and SH-SY5Y cells treated with 500 μL hops extracts, XAN 10–100 μM, and/or 50 μM H_2_O_2_ for 30 min or 24 h.	Hops reversed oxidative stress-induced cell death; XAN did not but modulated adenosine receptors.	Modulation of adenosine A1 and A2 receptors.	[51]
Investigate how XAN and related prenylflavonoids interact with Aβ1-42 oligomers to modulate amyloid aggregation.	Structural and molecular analysis of prenylflavonoids with Aβ1-42 and evaluation of anti-amyloidogenic properties at molecular level. XAN is tested with 0.5 μM, 0.15 mM and without defined concentrations.	XAN strongly inhibited Aβ1-42 aggregation at low concentrations, stabilized amorphous aggregates, and prevented β-sheet fibril formation.	XAN forms stable complexes with Aβ1-42 oligomers via conformational flexibility, redirecting aggregation toward less toxic forms.	[52]
Investigate the effect of XAN on the adenosinergic pathway, potentially involved in AD pathology.	Cell culture study using C6 (rat glioma) and SH-SY5Y (human neuroblastoma) cells treated with 10 µM XAN.	XAN may protect against AD by enhancing A1 receptor-mediated inhibition of excitotoxicity and modulating adenosine metabolism.	XAN increased A1 receptor levels. No effect on A2A receptors or adenylate cyclase activity. CD73 (5′-nucleotidase) significantly decreased in C6 cells.	[53]
Investigate the neuroprotective effects of XAN in rats with MCAO-induced cerebral ischemia.	Rats were treated with XAN (0.2–0.4 mg/kg, i.p.) 10 min before MCAO.	Reduced infarct size and improved neurobehavior.	Suppressed HIF-1α, TNF-α, iNOS, and caspase-3 expression. Inhibited platelet aggregation and hydroxyl radical formation.	[54]
Investigate the neuroprotective effects of XAN on age-related brain inflammation and apoptosis.	Male SAMP8 mice (aging model) treated with XAN (1 or 5 mg/kg/day) for 30 days and comparisons made with young/old SAMR1 controls.	Anti-inflammatory and anti-apoptotic effects, and preservation of synaptic markers suggest XAN protects against aging-induced neurodegeneration.	XAN reduced expression of pro-inflammatory (TNF-α, IL-1β) and pro-apoptotic (AIF, BAD, BAX) markers. A dose of 5 mg/kg restored synaptic proteins (BDNF, synapsin, and synaptophysin).	[55]
Investigate the neuroprotective effects of XAN in ischemic stroke.	Male Sprague Dawley rats were divided into sham, MCAO, and XAN (0.4 mg/kg, i.p.) groups (n = 12). MCAO and XAN groups underwent 60 min middle cerebral artery occlusion with 24 h reperfusion; sham rats had surgery without occlusion.	XAN reduced brain infarct size, improved neurological function, and reduced oxidative stress and neuronal apoptosis.	Inhibition of p38-MAPK phosphorylation and activation of Nrf2-mediated antioxidant response.	[56]
Assess the protective effects of XAN against glutamate-induced excitotoxicity.	Male Sprague Dawley rats were randomly assigned to 6 groups: control, XAN 10 or 50 mg/kg, kainic acid (KA) 15 mg/kg, and XAN + KA. XAN was given i.p. 30 min before KA. Seizures were monitored for 4 h.	XAN reduced KA-induced seizures, glutamate elevation, and neuronal death in the CA3 region of the hippocampus.	XAN upregulated mitochondrial fusion protein Mfn-2 and antiapoptotic Bcl-2, inhibited Apaf-1 and caspase-3 activation, preserving mitochondrial function and promoting neuron survival.	[57]
Evaluate the protective effects of XAN against LPS-induced depressive-like behaviors via neuroinflammation and oxidative stress pathways.	Mice were pretreated with XAN (10/20 mg/kg) before LPS (1 mg/kg) induction, behavior tests.	XAN reduced neuroinflammation.	XAN activates Nrf2/HO-1 antioxidant pathway and inhibits NF-κB signaling.	[58]
Assess whether XAN improves memory.	Male APP/PS1 and 10 C57BL/6J mice, divided into 5 groups: CON, APP/PS1, APP/PS1 + NAC (100 mg/kg), APP/PS1 + XAN-L (30 mg/kg), APP/PS1 + XAN-H (90 mg/kg) for 6 days/week for 2 months.	XAN improved memory performance, increased SOD, reduced IL-6 and IL-1β, decreased hippocampal Aβ deposition, and promoted autophagy and anti-apoptotic signals. It shows anti-inflammatory and antioxidant effects.	Activation of mTOR/LC3 (autophagy) and Bax/Bcl-2 (apoptosis inhibition) pathways.	[59]
Examine how XAN modulates gut microbiota and cognitive function in APP/PS1 mice as a potential AD treatment.	APP/PS1 and C57 WT mice; preventive (2-month-old) and therapeutic (6-month-old) studies; 4 groups per study: WT + corn oil, WT + XAN (5 mg/kg), APP/PS1 + corn oil, APP/PS1 + XAN (5 mg/kg) for 90 days.	XAN shows protective role in regulating gut microbiome composition and metabolism in an animal model of AD.	-	[60]
Investigate how XAN repairs cognitive impairment caused by estrogen deprivation.	Thirty C57BL/6J female mice divided in 5 groups: sham-operated, OVX + saline (vehicle), and OVX + xanthohumol at 10, 25, or 50 mg/kg. XAN was administered intraperitoneally every 2 days.	XAN improved learning and memory in OVX mice; miR-532-3p was downregulated, and Mpped1 expression was restored; overexpression of Mpped1 improved cognition.	XAN inhibits miR-532-3p, thereby restoring Mpped1 expression in the hippocampus to reverse cognitive impairment.	[61]
Evaluate the neuroprotective effects of HLE and XAN against iron-induced nerve damage.	Male C57BL/6 mice were randomly assigned to 8 groups: control, iron dextran plus vehicle, iron dextran + humic acid (0.1 mg/kg/day), iron dextran + N-acetylcysteine (100 mg/kg/day), iron dextran + low-dose hawthorn leaf extract (3 g/kg/day), iron dextran + high-dose hawthorn leaf extract (9 g/kg/day), iron dextran + low-dose XAN (30 mg/kg/day), and iron dextran + high-dose XAN (90 mg/kg/day).	HLE and XAN improved memory, reduced oxidative stress, and increased antioxidant enzyme activity.	Activation of AKT/GSK3β and Nrf2/NQO1/HO-1 signaling pathways.	[62]
Explore how XAN prevents memory loss in AD.	APP/PS1 mice: wild-type, Alzheimer’s disease model (AD), or AD + XAN, 0.5 mg/kg groups for 90 days.	Inhibited excitotoxicity, enhanced mitochondrial function, regulated systemic glutamate.	Reduced excitatory receptor expression, boosted ATP and mitophagy, lowered glutamate levels.	[63]
Explore the preventive and therapeutic mechanisms of XAN in AD using metabolite analysis.	APP/PS1 mice. Animals received XAN (5 mg/kg, gavage, every other day, 90 days), corn oil (vehicle), or memantine (5 mg/kg, positive control).	XAN improved cognition in older mice.	XAN influences cognition via endogenous metabolite regulation.	[64]
Identify therapeutic targets of different XAN doses in early Alzheimer’s using proteomics and microbiomics.	APP/PS1 mice Treated with 0.5 mg/kg and 5 mg/kg XAN.	0.5 mg/kg XAN improved memory, neurogenesis and reduced inflammation.	XAN modulates neurodegeneration pathways and gut microbiota. Low dose may optimize brain–gut signaling and cognitive benefits.	[65]
Improve oral bioavailability and therapeutic efficacy of XAN for AD.	AD was induced by AlCl_3_ (100 mg/kg, p.o.) for 56 days in rats. Treatments: XAN (30 mg/kg), donepezil (1 mg/kg), and XAN NLCs (15, 30 mg/kg), with corresponding controls. Behavioral tests were performed on days 56, 70, and 84.	Enhancements in cognitive and motor performance, along with decreased levels of AChE, Aβ, oxidative stress, and neuroinflammation.	-	[66]

**Table 4:** Clinical trials on β-caryophyllene and xanthohumol (clinicaltrials.gov).

Study Overview	Phase	Status	Trial ID
Xanthohumol			
Determine the pharmacokinetic profile of xanthohumol after oral intake.	Not applicable	Completed	NCT01367431
Evaluate the capability of xanthohumol to prevent damage of DNA and reduce oxidative stress.	Phase 1	Completed	NCT02432651
Determine the effect of xanthohumol on metabolic syndrome progression.	Not applicable	Unknown	NCT03561116
Determine the effect of xanthohumol and iso-alpha acids on the immune response of healthy participants (placebo-controlled crossover study).	Not applicable	Completed	NCT04847193
Determine the biological effect of xanthohumol exposure and its metabolism by intestinal microorganisms (double-masked, placebo-controlled, randomized clinical trial).	Phase 1	Ongoing	NCT03735420
Evaluate the safety and tolerability of xanthohumol and its effect on adults with Crohn’s disease (double-masked, placebo-controlled, randomized clinical trial).	Phase 2	Ongoing	NCT04590508
Determine the effect of xanthohumol on clinical course, inflammatory response and outcome of patients with COVID-related acute respiratory failure.	Early phase 1	Suspended	NCT05463393
Evaluate the rate and extend of the plasma presence of xanthohumol and micelle-incorporated xanthohumol in healthy men and women.	Not applicable	Completed	NCT05524714
Evaluate the capability of xanthohumol to reduce the inflammatory response in patients with septic shock (randomized, double-blind, placebo-controlled study).	Phase 2	Ongoing	NCT06225258
Evaluate the effect of xanthohumol on the severity of symptoms and duration of viral infections (placebo-controlled study.	Not applicable	Ongoing	NCT06286657
Investigate how xanthohumol affects the resting energy expenditure and substrate oxidation in healthy women.	Not applicable	Completed	NCT05711212
Evaluate the effect of iso-alpha acids and xanthohumol on the immune response in overweight patients.	Not applicable	Ongoing	NCT06745102
β-caryophyllene			
Determine the pharmacokinetics of β-caryophyllene and to evaluate its analgesic effect on thermal pain (randomized, placebo-controlled, double-blind study).	Phase 2	Withdrawn	NCT04794205
Evaluate the effectiveness of combined treatment of oxygen–ozone injection and topical patches containing cannabidiol and β-caryophyllene, compared to oxygen–ozone injection alone in patients with neck pain (randomized, controlled study).	Phase 3	Ongoing	NCT06099171
Evaluate the analgesic effect of 20% topical cream containing β-caryophyllene alone or in combination with 0.025% capsicum oleoresin, in patients with knee osteoarthritis-induced pain (randomized, double-blind, placebo-controlled crossover trial).	Phase 2	Completed	NCT03152578

## Data Availability

No new data were created or analyzed in this study. Data sharing is not applicable to this article.

## Abbreviations

The following abbreviations are used in this manuscript:

XAN	Xanthohumol
NDDs	Neurodegenerative diseases
AD	Alzheimer’s disease
ALS	amyotrophic lateral sclerosis
BCP	β-Caryophyllene
BBB	Blood–brain barrier
CB2	Cannabinoid receptor 2
HLB	Hydrophilic lipophilic balance
MβCD	Methyl-β-cyclodextrin
MS	Multiple sclerosis
NO	Nitric oxide
PLGA	Poly(lactic-co-glycolic) acid
PEG	Polyethylene glycol
PCL	Poly-caprolactone
PPARγ	Peroxisome proliferatoractivated receptor-γ
PVA	Polyvinyl alcohol
TC	Trans-caryophyllene
TNBC	Triple-negative breast cancer
CD	Cyclodextrin
DDS	Drug-delivery system
TNF-α	Tumor necrosis factor α
IL-6	Interleukin 6
DSC	Differential scanning calorimetry
PXRD	Powder x-ray diffraction
FDA	Food and Drug Administration
